# Health System Barriers to Provider-Initiated HIV Testing and Counselling Services for Infants and Children: A Qualitative Study From 2 Districts in Njombe, Tanzania

**DOI:** 10.24248/EAHRJ-D-16-00359

**Published:** 2017-07-01

**Authors:** Gasto Frumence, Sirili Nathanaeli

**Affiliations:** Muhimbili University of Health and Allied Sciences, Dar es Salaam, Tanzania

## Abstract

**Background:**

There is low utilisation of provider-initiated HIV testing and counselling (PITC) services for infants and children under 5 years old in many low- and middle-income countries including Tanzania. Studies have shown that various factors contribute to low use of PITC, includes the unavailability of the polymerase chain reaction (PCR) test and other specialised techniques for testing children less than 18 months old as well as the reluctance of some parents and caregivers to undertake HIV testing for their children because of the fear of stigma associated with HIV/AIDS. This study explored health system barriers at the district and community levels affecting the provision of PITC for infants and children under 5 in Tanzania using a case study of 2 districts.

**Methods:**

A qualitative study was conducted in 1 urban and 1 rural district in the southern part of Tanzania. In-depth interviews, focus group discussions, and a desk review of documents were used to obtain the information. Respondents were purposively enrolled in the study and thematic analysis was used to generate findings.

**Results:**

Provision of PITC services faces a number of district-level health system barriers, including lack of adequate health staff in health facilities both in number and skills, lack of adequate infrastructure, and erratic shortage of supplies. At the community level, community members’ low understanding about the importance of PITC services as well as existing stigma associated with HIV/AIDS have constrained the provision of PITC services.

**Conclusion:**

This study concludes that for effective implementation of PITC, the health system should strengthen health facilities through training of service providers on PITC, deploying adequately skilled health workers, supplying sufficient medicines and other supplies, and promoting health campaigns focused on educating community members about the importance of early HIV testing for infants and children under 5.

## INTRODUCTION

Worldwide, approximately 36.7 million people were living with HIV/AIDS in 2016; of these, about 2.1 million were children and adolescents under 15 years of age.^1^ In 2014 alone, it was estimated that 190,000 children were newly infected with HIV throughout Africa and about 3% of deaths in children under 5 years old were attributed to AIDS.^2^ Most of these children were infected through vertical transmission, from mother to child, which accounts for more than 90% of deaths in children under 5 in sub-Saharan Africa.^3^ Despite various global and national efforts to scale up HIV prevention, care, and treatment, services have not been fully implemented for infants and children who have been exposed or infected with HIV.^4^ The World Health Organization (WHO) recommends offering routine provider-initiated HIV testing and counselling (PITC) to persons attending health care facilities as a standard component of medical care.^5^ Early diagnosis of HIV infection in infants (0–12 months of age)^6^ and children under 5 is important for timely initiation of treatment with antiretroviral therapy.^7,8^

Several studies have reported a number of barriers to testing children for HIV, including unavailability of the polymerase chain reaction (PCR) test and other specialised techniques in testing children less than 18 months old in many health facilities. The stigma associated with HIV/AIDS also contributes to the reluctance of some parents and caregivers to undertake HIV testing for their children.^9^ Other barriers include poor linkages with prevention of mother-to-child transmission programmes and subsequent missed opportunities for testing during postnatal and child health care, challenges of providing virological testing required for early infant diagnosis (eg, RNA or DNA PCR), limited paediatric expertise amongst health care providers, and the prioritisation of adult treatment and subsequent lag in availability of paediatric antiretroviral therapy dosages.^10–14^

In low- and middle-income countries, late HIV diagnosis and delayed care interventions result in high mortality rates amongst HIV-infected children.^4^ Tanzania adopted PITC in 2007 as one of the strategies for increasing access to HIV care in the country.^15^ PITC is a system whereby HIV testing and counselling are provided to all patients attending a health facility, regardless of the reason for their visit.^4^ Many countries in sub-Saharan Africa, including Tanzania, have developed and scaled up access to early infant HIV virological testing, particularly using dried blood spot testing.^16–18^ These blood spots are usually collected from health facilities and sent to referral hospitals’ laboratories. However, this initiative faces several difficulties, such as the long delay between blood collection, the resulting large proportion of parents and caregivers who fail to return for the results, and the high number of HIV-infected infants who are lost to follow-up.^3^ In Tanzania, little is known about the barriers to early HIV diagnosis of infants and children under 5. Therefore, this study aimed to explore health system barriers at the district and community levels for early diagnosis of HIV in infants and children under 5 in Tanzania.

## METHODS

### Study Design

We used a cross-sectional exploratory study design to explore health system barriers at the district and community levels for HIV testing amongst infants and children and analysed results using a phenomenological approach. According to Lopez and Willis,^19^ a phenomenological approach seeks to describe the meanings embedded in the human experience and common life practices. Furthermore, this approach helps to explore the individual experiences as well as common characteristics and differences between groups of participants.

### Study Setting

This study was carried out in Njombe Region located in the Southern Highland Zone. This zone was purposefully chosen because it has the highest HIV prevalence in the country, and, within it, the Njombe Region was selected due to its high HIV prevalence – 14.8% compared with the national HIV prevalence of 5%. Within the region, the Njombe and Makete districts were chosen to present the rural (Makete) and urban (Njombe) districts. The 2 districts also had high HIV prevalence in the region: 14.7% in Makete and 15.34% in Njombe District.^20^

### Study Population

The study population consisted of health service providers at regional and district hospitals and health centres where HIV testing for infants and children is done, parents and caregivers with infants and children under 5, and women who recently gave birth (in the past 42 days to 18 months).

### Sampling Techniques and Recruiting Plans

We used the purposive sampling method to select 1 zone with high HIV prevalence amongst the 7 geographical zones. Within this zone, we purposively selected 1 region with high HIV prevalence, along with 2 districts with high HIV prevalence (1 urban and 1 rural). Within each district, the district hospital and 1 health centre from a randomly selected ward, amongst the wards with health centres, were included.

We also used purposive sampling to select health service providers, including the district hospital secretary, health facility in-charges, heads of care and treatment clinics, heads of laboratories, and heads of reproductive and child health units.

A convenience sampling was applied to select parents and caregivers with children under 5 and recently delivered mothers for conducting focus group discussions (FGDs). Recently delivered mothers included all women who had given birth in the previous 42 days (6 weeks) to 18 months. The choice for this post-puerperium period was based on cultural practices that hinder contacting mothers within the first 6 weeks after delivery. The upper limit was based on the fact that HIV antibody confirmatory testing in children is reliable after 18 months of age.^21^ Community health workers supported the process of identifying recently delivered mothers in their communities.

### Data Collection Methods

A total of 18 face-to-face in-depth interviews were conducted with the district hospital secretary and all in-charges and heads of sections of selected health facilities. In addition, a total of 4 FGDs were conducted to gather information from families—2 FGDs with recently delivered mothers and 2 FGDs with parents and caregivers of infants and children under 5. Both in-depth interviews and FGDs aimed to explore district- and community-level health system barriers to HIVPITC services for infants and children under 5. Data collection was stopped after 18 interviews and 4 FGDs after reaching the point of data saturation when no new themes could be found in the participants’ statements. In-depth interviews and FGDs were conducted in health facility and village offices and lasted for 60 and 90 minutes, respectively. These offices provided adequate privacy without interference from people who were not participating in the study. The in-depth interview and FGD guides were developed, pre-tested, and revised before being used to collect data for this study.

A desk review focused on key strategic documents and guidelines on PITC services to clarify the themes that emerged from the in-depth interviews and FGDs and to analyse the study findings in the context of Tanzania. This triangulation of data collection techniques – namely, the in-depth interviews with key informants, FGDs with recently delivered mothers, as well as desk reviews – helped to ensure the credibility of the study findings.^23^

### Research Team

The first (GF) and second (SN) authors and 2 research assistants were involved in collecting data for this study. GF and SN were responsible for conducting in-depth interviews and the 2 research assistants were responsible for conducting FGDs. It is worth noting that no relationship existed between the research team and study participants before their engagement in this study.

### Quality Control and Data Management

Trained research assistants listened to the audio-recorded information every day to check the accuracy and sounds, and, where possible, to take corrective measures to rectify any missing information. Moreover, periodic team meetings with the principal investigators and research assistants were held to review progress.

### Data Analysis

In this study, the research team used a thematic analysis method. Data were coded without necessarily fitting them into a pre-existing coding frame or the researcher's analytic preconceptions.

The 2 research assistants transcribed the data and coded it manually. The researchers then analysed the data manually, examined the transcripts and field notes, and reviewed documents. Using an inductive approach, the researchers identified concepts that emerged and were strongly linked to the data themselves^24^ and that described the phenomenon under investigation.^25^ These concepts were further analysed to identify their similarities and differences; subsequently, they were grouped together to form more precise categories that were later organised into themes based on the research objective. Manual analysis was done for documents to clarify the themes and hence understand the emerging themes in the context of Tanzania.

### Ethical Consideration

Ethical clearance to conduct this study was obtained from the Medical Research Coordinating Committee through the National Institute of Medical Research. After obtaining ethical approval, permission to carry out the study was obtained from the regional and district officials. The district officials introduced the research teams to the wards and villages. Written informed consent was obtained from all the respondents. The consent form contained information on the objective of the study, procedures of the study, rights to withdraw, confidentiality, and feedback of results. The instruments were translated into the local language, Kiswahili, and interviews and FGDs were held in private.

## RESULTS

### Barriers at the District Level

A total of 18 interviews were conducted with service providers, namely the district hospital secretary, in-charges of health facilities, heads of care and treatment clinics, laboratories, and reproductive and child health units from the selected health facilities.

The research team conducted a thematic analysis of the data. Seven themes emerged showing institutional barriers hindering the provision of PITC services at health facilities: a shortage of tools and supplies for providing testing and counselling services, a shortage of health workers at the facility level, overcrowding of clients, inadequate number of trained service providers on PITC, low motivation amongst workers, inadequate space for providing PITC, and harsh language used by some service providers.

#### Shortage of Tools and Supplies for Providing Testing and Counselling Services

Our findings showed that the district health system lacks adequate tools and supplies for HIV testing. Key informants reported a shortage of diagnostic kits and buffers (reagents) as a common barrier at most of the health facilities visited in the 2 districts. In some places, even consent forms were in low supply. Key informants reported that this shortage of tools and supplies discourages clients from coming to facilities where they do not receive adequate services.

Sometimes we have many clients until we run out of diagnostic kits or reagents … we may stay for a month or more without kits or reagents … —Key informant, district hospital

Another respondent said:

The government should first address the challenge of insufficient diagnostic kits and reagents if it is interested to ensure HIV test is done to all eligible clients including children … for us we play our part if there are adequate resources. —Key informant, district hospital

#### Shortage of Health Workers at the Facility Level

Findings from most of the key informants show that there was a shortage of staff to deliver the PITC services in many health facilities in the 2 study districts.

We have a severe shortage of staff here … and this hospital although is located at a town council we are doing a job like a regional hospital … some of our clients come from areas that are outside our catchment area … —Key informant, health centre

Another study informant expressed the following:

Sometimes we are asked to postpone our annual leave because of shortage of workforce…. This is a major challenge. —Key informant, health centre

A midterm evaluation of the 2008–2013 Human Resources for Health Strategic Plan in 2013 showed that the country had only 44% of the health workers required to serve the total population of the country.^22^ This lack of availability was asymmetrically distributed between rural and urban areas; although rural areas contained more than 75% of the total population, they had only 55% of the health workers needed to serve the population.^23^

#### Overcrowding of Clients

Key informants reported overcrowding of clients in some health facilities, which is caused mainly by the shortage of staff. The problem is worse in some health facilities, especially those based in town centres where other clients come from nearby districts and even regions.

Sometimes we are facing a problem of overcrowding of clients to the extent that a service provider cannot remember to initiate testing and counselling service for the infants or even children under 5 … because we do not even get time for lunch break … —Key informant, district hospital

One respondent also stated that the problem of overcrowding of clients also exist in health facilities situated in rural areas.

You know … because of shortage of staff in this facility, in mostcases youfind us overwhelmedwith clients makingitdifficult to provide all services … —Key informant, health centre

#### Inadequate Number of Trained Service Providers on PITC

Key informants in all districts were concerned with the problem of having service providers who are not trained on PITC or exposed to counselling services. This situation was described as a barrier toward effective provision of PITC services in some of the health facilities as expressed below by key informants:

… it is not easy for the facility to provide effective provider-initiated testing and counselling services when there is not enough staff trained for this service or oriented for such services … —Key informant, health centre

… job training has never been enough. Staff must attend at least short courses to gain more knowledge and skills to initiate counselling and HIV testing services for their clients … —Key informant, district hospital

#### Low Motivation Amongst Workers

Findings from this study revealed that the majority of health workers in departments where PITC is delivered had low motivation due to a number of factors. Some of the factors included work overload, lack of regular refresher courses, inadequate supplies for rendering services, and low remuneration. One of the key informants had this to say:

We are only 2 in this department, today my colleague is sick. I have so many clients as you saw … I have been here for more than 5 years but I have not attended even a 1-week seminar … I just work because I have no alternative work, but I am really tired … —Key informant, district hospital

Health workers in public health facilities are experiencing a heavy workload, which contributes to low motivation, as expressed by a study participant:

Work overload is greatly demoralising me. The government should recruit more staff if it wants to improve the provision of services … —Key informant, district hospital

#### Inadequate Space for Providing PITC

It was reported that since PITC is provided in all departments, a shortage of rooms in many health facilities in all districts reduces the level of privacy to the clients. In 1 of the districts, key informants said that some of the doctors’ consultation rooms accommodate 2 or more clinicians who provide services at the same time. In this regard, a key informant expressed the following:

… availability of rooms is a barrier; we have very few buildings here and some of the rooms accommodate more than 1 doctor … now in such scenario privacy is always compromised, this may limit others from up-taking PITC services … —Key informant, district hospital

As you can see here, we have very limited space here. How can you ensure privacy when interacting with clients…? —Key informant, health centre

#### Harsh Language Used by Some Service Providers

Another barrier for the effective provision of PITC services is that some service providers use harsh language when interacting with clients. FGD participants described harsh language as a demotivating factor for clients to accept PITC services in some of the facilities.

Some service providers treat clients with negative attitude and they use harsh language to the extent that some patients hesitate to undertake the requested HIV testing … —Recently delivered mother, FGD

There are few service providers who are not user friendly … but we have no alternative health facility … no option. —Recently delivered mother, FGD

### Barriers at the Community Level

Study participants were asked to give their views about community barriers hindering the use of PITC in their areas.

The analysis of the findings generated 3 main themes: low understanding of community members about the importance of HIV testing for children, reluctance of male partners to accompany their partners to the heath facility, and stigma associated with HIV/AIDS.

#### Low Understanding of Community Members About the Importance of HIV Testing for Children

Study participants mentioned that some community members, both men and women, have low understanding about the importance of HIV testing for infants and children under 5. The FGD participants reported a lack of health promotion campaigns about the need for HIV testing for children.

… majority of us including our partners are not aware of the importance of HIV testing for our infants. Maybe there should be more health campaigns to sensitise the community members about HIV testing to our children. —Recently delivered mother, FGD

#### Reluctance of Male Partners to Accompany Their Partners to the Heath Facility

Key informants reported that the provision of PITC could be more effective if male partners could accompany their female partners when visiting health facilities. They noted that men were reluctant to attend reproductive and child health clinics, making it difficult to initiate PITC services to women only. Low male involvement makes counselling difficult when disclosing results to the partner, as reported by a key informant:

… most of the time you have the woman herself visiting a health facility, the man is not there … it is very hard to counsel the woman to disclose her status or child status to her partner … It could be easy if they were together … —Key informant, health centre

Recently delivered mothers in FGDs reported similar findings that low male involvement in reproductive and child health services hinders women's efforts in accessing PITC services. This concern was well narrated by 2 recently delivered mothers:

… most men here do not want to accompany you to the clinic … they tell you when you are tested it is the same as I have also been tested … it therefore becomes difficult to tell him when you are tested HIV positive, as sometimes he will beat you claiming that you are the source … —Recently delivered mother, FGD

If our husbands could escort us to the clinic, they could participate in the counselling session and HIV testing and accepting the results could be easy to both of us … —Recently delivered mother, FGD

#### Stigma Associated With HIV/AIDS

Recently delivered mothers who participated in the FGDs said the main barrier for them to accept PITC is stigma associated with HIV/AIDS in the community. Most of the women are afraid of how fellow community members would look at them when they know they are HIV positive. They reported further that they fear being segregated, a situation that would affect them psychologically. Recently delivered mothers who participated in the FGDs expressed that stigma still persists in the community because some of the community members have low knowledge about HIV/AIDS.

Stigmatising HIV-positive people is a result of low level of knowledge about HIV/AIDS, provision of HIV education should be an ongoing intervention … —Recently delivered mother, FGD

Another recently delivered mother said that stigma associated with HIV/AIDS is accelerated by unethical health workers who sometimes disclose a patient's HIV-positive diagnosis to community members.

Other service providers announce that someone is HIV positive. This is why some people are hesitant to easily accept HIV test … —Recently delivered mother, FGD

## DISCUSSION

This study aimed to identify health system barriers at the district and community levels that hinder the provision of PITC for infants and children under 5. At the district level, the study found a number of barriers hindering the provision of PITC services, most of which were found at the health facility level. Shortages of adequate service providers, lack of PITC training to the available service providers, and lack of adequate diagnostic kits were amongst the leading barriers. On one hand, shortages of human resources, trained personnel, and diagnostic kits for PITC are contrary to the Tanzania National HIV/AIDS Policy, which emphasises the availability and accessibility of HIV testing and counselling services to all people in the country.^26^ On the other hand, the national guidelines for HIV counselling and testing in Tanzania stipulate clearly that PITC could only be effective if there are adequate service providers who are trained in PITC.^15^

A study on knowledge, attitudes, and acceptability to PITC from the patients’ perspectives in Moshi and Rombo districts in Tanzania revealed similar findings – that lack of adequate human resources in the health facilities hinder the provision of PITC services.^27^ A similar study conducted in Mbeya Region in Tanzania examined perceived barriers and attitudes of health care providers toward PITC and revealed that lack of trained staff may affect the provision of PITC services.^28^ In Zimbabwe, a study on barriers to PITC for children in a high HIV-prevalence setting reported that health workers lacked training on counselling for children and guardians, which affected the provision of PITC services. This study emphasised that service providers should be trained on PITC, paying particular attention to counselling male guardians, who are less likely to engage with health care services.^29^ In Flanders, Belgium, Manirankunda and colleagues reported that lack of expertise in sexuality counselling is a challenge for PITC services to sub-Saharan African migrants.^30^

In this study, we found that lack of private space and shortages of PITC guidelines for paediatrics, rapid test instruction manuals, and rapid test kits hindered the effective implementation of PITC services. A study in Malawi reported similar findings showing that lack of privacy in the clinics limited uptake of PITC services for infants.^31^ Lack of adequate resources required to implement PITC has also demoralised service providers, thus constraining effective provision of PITC services. A number of studies^27,32,33^ have discussed the importance of incentives for health workers, such as good salaries and training, to increase motivation and commitment and ultimately improve performance. This implies that lack of incentives amongst health workers leads to poor work performance. Lack of HIV testing kits or shortages of kits in various hospital laboratories has been reported elsewhere including Zimbabwe.^29^

Olmen and colleagues suggest that for the health system to contribute significantly toward improving the performance of health service delivery, there must be an adequate and competent health workforce, good governance, and sufficient resources.^34^ Furthermore, according to WHO, the government, through health sectors, needs to put in place health financing mechanisms that can ensure access to health services for all people, which in turn help to protect community members from disastrous health expenditure.^35^

At the community level, understanding and acceptance of parents and caregivers to undertake HIV testing for their children were mentioned as important factors that may influence the provision of PITC services in the study area. Similar findings were also reported from Zimbabwe, showing that some caregivers declined or did not give consent when service providers asked them to undertake HIV test for the children.^29^

Furthermore, this study shows the surprising finding that stigma associated with HIV/AIDS persists in the community, despite that it has been more than 3 decades since the implementation of interventions focused on reducing stigma. In this study it was reported that HIV/AIDS-related stigma hinders the provision of PITC services. These findings are in agreement with other studies,^27,36^ which underscores that HIV stigma is a major barrier to implementation of HIV interventions in communities, including PITC services.

Moreover, this study found that low male involvement in attending reproductive, maternal, and child health services has limited the provision of PITC services in various health facilities. Because men are still dominant in making decisions pertaining to various household or family matters in most communities in Tanzania, their low involvement in reproductive and child health has a substantial effect on the uptake of PITC services in the community. A number of studies^37–39^ have pointed out a similar barrier indicating that use of HIV testing and counselling services in most health facilities is low because men do not accompany their female partners to the health facilities.

## CONCLUSION

This study concludes that the provision of PITC services at the district level faces a number of barriers, including lack of trained service providers, inadequate number of health workers, lack of adequate space, and shortages of HIV testing kits. At the community level, the provision of PITC services is constrained by lack of understanding and acceptance amongst parents and caregivers to undertake HIV testing for their children, low male involvement in seeking PITC services, and persistence of stigma associated with HIV/AIDS in the community. The study underscores that for the health system to achieve the desired goals of improving PITC services, the government needs to strengthen district- and community-level health systems through recruiting and deploying adequate skilled health workers, training service providers on PITC, mobilising adequate financial resources, and supplying sufficient medicine and other medical supplies. The government should also play an active stewardship role to ensure that the district health system implements interventions to increase awareness and understanding of the importance of PITC services and to reduce or eliminate stigma associated with HIV/AIDS in the community.
